# Demographic patterns and trends in Central Ghana: baseline indicators from the Kintampo Health and Demographic Surveillance System

**DOI:** 10.3402/gha.v5i0.19033

**Published:** 2012-12-20

**Authors:** Seth Owusu-Agyei, Obed Ernest A. Nettey, Charles Zandoh, Abubakari Sulemana, Robert Adda, Seeba Amenga-Etego, Cheikh Mbacke

**Affiliations:** 1 Kintampo Health Research Centre, Kintampo, Ghana; 2 The Secretariat, INDEPTH Network, Accra, Ghana

**Keywords:** Kintampo, demographic surveillance, population, Ghana, demographic rates, fertility, mortality, migration

## Abstract

**Background:**

The dearth of health and demographic data in sub-Saharan Africa from vital registration systems and its impact on effective planning for health and socio-economic development is widely documented. Health and Demographic Surveillance Systems have the capacity to address the dearth of quality data for policy making in resource-poor settings.

**Objective:**

This article demonstrates the utility of the Kintampo Health and Demographic Surveillance System (KHDSS) by showing the patterns and trends of population change from 2005 to 2009 in the Kintampo North Municipality and Kintampo South districts of Ghana through data obtained from the KHDSS biannual update rounds.

**Design:**

Basic demographic rates for fertility, mortality, and migration were computed by year. School enrolment was computed as a percentage in school by age and sex for 6–18 year-olds. Socio-economic status was derived by use of Principal Components Analysis on household assets.

**Results:**

Over the period, an earlier fertility decline was reversed in 2009; mortality declined slightly for all age-groups, and a significant share of working-age population was lost through out-migration. Large minorities of children of school-going age are not in school. Socio-economic factors are shown to be important determinants of fertility and mortality.

**Conclusion:**

Strengthening the capacity of HDSSs could offer added value to evidence-driven policymaking at local level.

Good data are essential for effective development planning and evaluation of development interventions. This is particularly true in resource-starved countries in sub-Saharan Africa where inappropriate resource allocation decisions tend to perpetuate poverty and under-development. The Millennium Declaration, signed in 2000 by 189 heads of state and government, is the main development framework for many less developed countries (LDCs). All signatories are expected to monitor and report annually on progress towards achievement of the targets set forth by the Millennium Development Goals (MDGs). The MDG framework puts data and evidence at the centre of the global effort to reduce poverty and promote economic and social development (1–4).

Because the major goal of development efforts is to satisfy the current and future needs of the population, demographic data are fundamental. The United Nations International Conference on Population and Development (ICPD) in Cairo 1994 recognised the important role that demography plays in socio-economic development by recommending the full integration of population factors into development strategies (5–7). In spite of this, vital registration systems in many countries in sub-Saharan Africa are undeveloped and their coverage is minimal. For instance, as of December 2003, regional coverage of death registration ranged from 100% in the European Region to less than 10% in the African Region (8). In Ghana, the Births and Deaths Registry (BDR) recorded an estimated 49% of births and 25% of deaths from 2000 to 2008. Some of the challenges to universal coverage of the BDR include: limited access to registration facilities, low public knowledge about the importance of registration, inability of the BDR to attract and retain highly qualified personnel (due to low remuneration and poor service conditions), and lack of logistics including accommodation, vehicles, requisite statistical software and programmes.

Therefore, the main sources of demographic data in sub-Saharan Africa are sample surveys such as the Demographic and Health Surveys and the censuses that are conducted, at best, every decade. The Demographic and Health Surveys, which have been conducted every 5 years in Ghana since 1988, have provided valuable nation-wide data on some demographic and health indicators (9, 10). However, they are of limited use for planning at the district levels because of the low sample size that they are usually based on, and the lack of any longitudinal approach; that is, although the survey might be repeated on a 5-year cycle, there is no intention to include the same individuals again. Furthermore, while censuses do provide data at the lowest administrative levels, scientists need to resort to models and other indirect measurement techniques in order to derive basic demographic indicators from census data. The data may be out-dated by the time they are ready for use.

Health and Demographic Surveillance Systems (HDSSs), especially those within the INDEPTH Network, are operating in places where routine vital registration systems are non-existent or poorly developed; constituting an alternative source that can produce timely and reliable vital-registration-like data at the district level (3, 11–14). The Kintampo Health and Demographic Surveillance System (KHDSS) is one such HDSSs, operating within the purview of the Kintampo Health Research Centre (KHRC). KHRC is one of three research centres established under the Ministry of Health in Ghana in the country's major ecological zones in the early 1990s. It provides regular updates on vital events such as pregnancies, births, deaths, and migrations (in and out) and covers the whole of the Kintampo North Municipality and the Kintampo South district located in the Brong-Ahafo Region of Ghana (4). The operations of a HDSS begin with a baseline census, followed by core activities involving monitoring of all entries into the dynamic resident population through births and in-migrations and all exits through deaths and out-migrations. This is shown as a conceptual framework of the HDSS operations in Fig. 1.

**Fig. 1:**
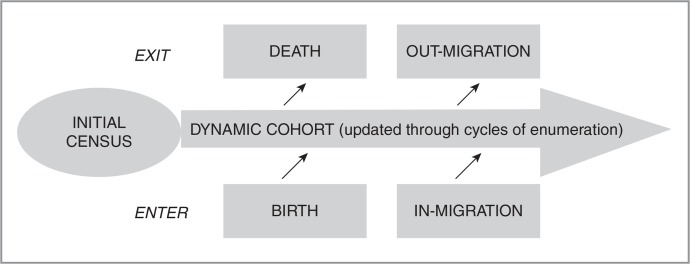
Conceptual framework for HDSS field operations.

This article sets out to present the basic demographic data collected from January 2005 to December 2009 as part of the HDSS activities in the Kintampo area. This comprises births, deaths and migrations. Health and Demographic information further to the core activities of the KHDSS includes socio-economics, household wealth, educational status, and causes of death that are routinely collected. In addition, basic patterns and trends of these events are highlighted. With the approach of the decision point for the achievement of the MDGs, the utility of HDSS cannot be over-emphasised (13).

## Methods

The data source for this article is the KHDSS, which was updated twice a year from January 2005 to December 2009 for each household, with the first update covering January to June and the second update July to December each year. Demographic events such as births, deaths and migrations are recorded during routine 6-monthly update visits. Furthermore, prominent community members act as community key informants (CKIs), recording all pregnancies, births and deaths that come to their attention. They are usually abreast with the day-to-day activities of the community. Thus, they are in a unique position to report on most of the events that occur. They are important for data quality in that they record events (e.g. pregnancies and early infant deaths), which may have occurred in-between update rounds, thus reducing the problem of under-reporting by fieldworkers visiting the household. They also partake in health outreach programmes, promotions and campaigns.

### The Kintampo Health and Demographic Surveillance Area

**Fig. 2:**
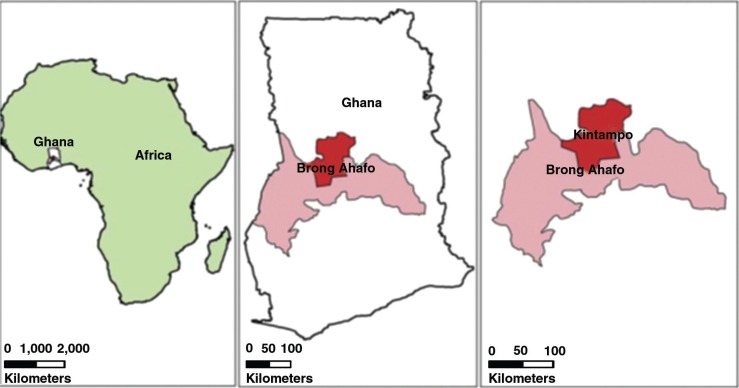
Kintampo Health and Demographic Surveillance Site, Ghana.

The Kintampo Health and Demographic Surveillance Area comprises the Kintampo North Municipality and Kintampo South District in the Brong-Ahafo Region of Ghana. It has a surface area of 7,162 km^2^, which is 18.1% of the total land area of the region. Its strategic location makes it the geographical centre of Ghana (see Fig. 2). Its vegetation is mainly of the forest–savannah transition type.

The KHDSS population is largely rural, constituting approximately 65% of residents, living in 29,073 households at the end of 2009. Only 25.8% of the total population has access to electricity, which is predominantly available in the urban areas. In 2008, approximately a third (32.9%) of households accessed water mainly from streams and rivers, 23% from hand-pumps and another 25.1% from closed wells. Water closet toilets were available to only 3.1% of the population in 2008 and these were exclusively in the urban areas. Pit latrines, the main toilet facility in the study area, are used by 59.1% of the population. Almost 40% of the population use open fields, which has implications for the health status of the population.

#### Births/fertility

Fertility was measured using crude birth rates (CBRs), age-specific fertility rates (ASFRs), general fertility rates (GFRs), and total fertility rates (TFRs) in order to demonstrate the levels, trends, and determinants within the demographic surveillance area. CBRs, ASFRs, and GFRs were computed by person-years observed. The computations and analyses were carried out in STATA by computing person-years for each woman from their dates of entry and exit by each year, which was added to their total person-years for that year. To be able to adequately estimate fertility, pregnancies for all women registered into the KHDSS are followed and their outcomes recorded by field staff or CKIs. All live births were then registered as individual members of the KHDSS, independent of subsequent survival. In the KHDSS updates, it was mandatory for fieldworkers to take note of any live births to visitors to the HDSS to alert the data collector in the next round to register the mother and her child as they become eligible. This procedure greatly improves the accuracy of birth dates of newly born babies and also increases reporting of births from eligible mothers with frequent in- and out-migrations. Pregnancy observation has also been used to increase the reporting of pregnancy outcomes.

#### Deaths/mortality

Deaths of all registered and eligible individuals were recorded, regardless of whether the death occurred at home or elsewhere. KHDSS collects more detailed information about the deaths to establish the cause of death through the use of verbal autopsies. Mortality data were extracted from the health and demographic surveillance database of the KHDSS and measured using crude death rates (CDRs) and age-specific death rates to demonstrate the levels, trends, and determinants within the demographic surveillance area. Infant and child mortality rates are highlighted in this article. Life expectancy is computed separately for males and females using life tables.

#### Migration

The KHDSS registers two forms of migrations: *external migration*, where the residence changes between a residential unit in the HDSS and one outside it and *internal migration*, where residence changes from one residential unit to another within the HDSS area. Recording internal migration is very important as it ensures the accuracy and validity of the KHDSS data. Internal migrations and migrants were identified through the KHDSS, and supporting information was collected in order to avoid double counting individuals and ensuring that their exposures to the social and physical environment are correctly apportioned. Migrations influence the registration of births and deaths. A death, for example would not be recorded for an individual who out-migrated before his or her death. The definition of migration refers to crossing the boundary of the demographic surveillance area, to bring the rates more in line with standard migration definitions. In- and out-migrations differ from internal moves in that the former refers to moves in and out of the HDSS area whilst the latter refers to moves within the HDSS area.

Migrations are considered as recurring events since an individual may make several migrations over time, both internally and externally. To maintain longitudinal integrity of data concerning individuals, KHDSS establishes whether an individual moving into the HDSS area from outside has previously been registered into the HDSS. The individual's current and previous records are matched so that he or she is not handled as a new individual in the system but as an individual under observation for several periods. New household occupants identified on the day of the staff visit are asked for the date of their arrival into the compound. Individuals who had not yet been resident for 3 calendar months were provisionally recorded on the back of the household register form for follow-up at the next visit by which time they will be qualified for registration as new in-migrants and given permanent identification numbers. Previously registered residents who were reported as no longer residing in the house on the day of the visit were provisionally recorded as being out of the area, but not considered to have migrated out until they had been away for at least three consecutive calendar months.

Gross migration rates (GMRs) and net migration rates (NMRs) by year were computed. GMRs were computed as the sum of all in- and out-migrations over the respective person-years for the sub-group of interest, whilst NMRs were calculated as the difference between in- and out-migrations over the sub-group population. These rates were also computed by age and sex.

#### Education

Updated longitudinal annual educational data are collected on all 6 years and above. Current/highest level of education reached is updated once every year for all of the eligible population. School enrolment was computed as a percentage in school by age and sex for 6–18 year-olds.

#### Household characteristics

Socio-economic status was derived by using Principal Components Analysis on household assets. For this analysis, households were categorised into quintiles. This is done separately for rural and urban households to control for place of residence, as urban households are often categorised as the richest whilst rural households are classified as poor.

## Results

### Population structure

The household is the basic social unit of interest in the KHDSS. This is largely comprised of one or more persons who may or may not be related by blood or marriage, but who accept one individual as their head. By the end of 2009, there were 29,073 households in 18,795 compounds. The median household size was five, with a third of all households headed by women. Approximately one-third of households in the KHDSS were in urban locations. The population under surveillance and growth rates for the years 2005–2009 is shown in Table 1.

**Table 1:** KHDSS population and growth, 2005–2009

Determinant	2005	2006	2007	2008	2009
Population (1st January)	116,855	116,845	119,261	122,846	129,279
New enumeration	886	–	–	4,240	635
Births	3,840	3,881	3,972	3,988	4,317
Deaths	962	933	988	937	923
Net migration	−5,698	−2,398	−1,375	−2,732	−184
Population (31st December)	116,845	119,261	122,846	129,279	134,970
Growth% (without new enumeration)	–	1.06	1.02	0.99	1.03

**Fig. 3:**
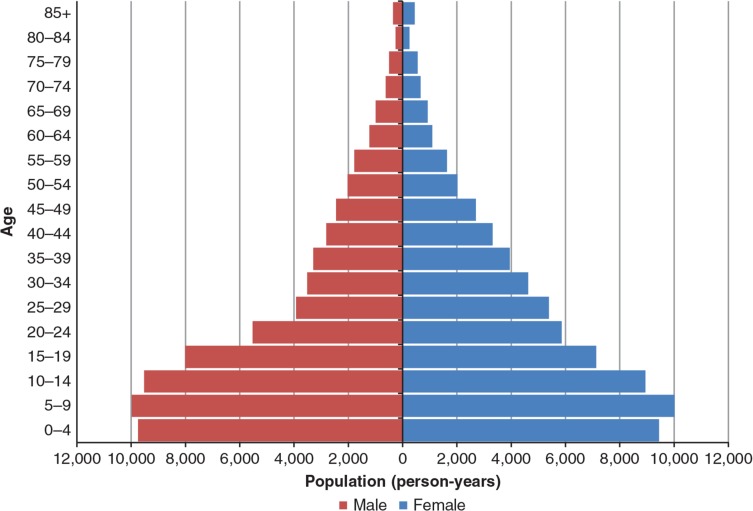
Population pyramid in person-years for KHDSS by 5 year age-groups and sex, 2009.

Without counting newly enumerated individuals, the KHDSS population grew by approximately 1% per annum. During the period, an additional 27 communities were brought under surveillance and some individuals in existing surveillance areas (but who were previously counted) were now brought into the system. The population pyramid for 2009 (in person-years) as shown in Fig. 3 indicate an under-enumeration of infants in the first year of life. The 2007–2009 population pyramids (not shown) improved significantly in terms of enumerations at age zero compared with that of 2005 and 2006 but was still lower than expected. The KHDSS population is very youthful, with 45.35% under the age of 15 years in 2009 and 5.39% aged 60 years and over in 2007.

### Components of population change

#### Fertility

**Fig. 4:**
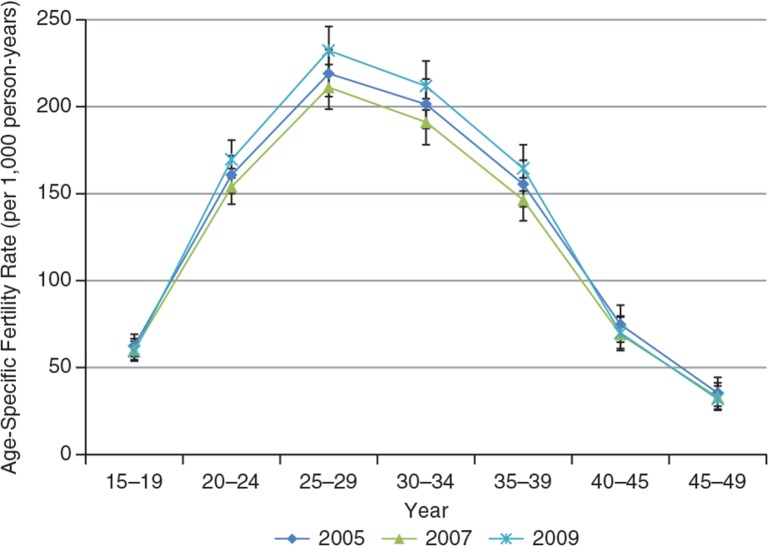
Age-specific fertility rates for KHDSS, 2005–2009.

The KHDSS recorded 19,998 births within the period 2005–2009, that is, approximately 4,000 births per year. The ASFRs of the KHDSS for these years are shown in Fig. 4.

The age-specific pattern of fertility indicated that the peak of fertility is within the age range 25–29 years. This suggests that fertility is in transition from pre-transition stage, where childbearing tends to be early. Teen fertility accounted for approximately 10% of total births for each year throughout the period, whilst births to women over 40 years accounted for approximately 7%. Fertility for women aged 25–29 dropped from 219 births per 1,000 person-years in 2005 to approximately 205 births per 1,000 person-years in 2008. Fertility dropped drastically after 39 years of age.

Table 2 shows some summary indicators of fertility in the KHDSS between 2005 and 2009. The TFR is the average number of children that would be born to a woman by the time she completed childbearing if she were to pass through all her childbearing years conforming to the ASFRs of a given year. By the end of 2008, a woman at the beginning of childbearing within the KHDSS would, on average, have 4.4 births by the end of her reproductive life. The year 2009 recorded a resurgence in fertility levels, after three consecutive years of decline from 2005.

**Table 2:** Fertility indicators for KHDSS, 2005–2009

Indicator	2005	2006	2007	2008	2009
GFR	137.13	131.98	130.45	127.06	137.86
TFR	4.55	4.38	4.33	4.23	4.70
CBR	33.16	31.98	31.41	30.57	31.84

The GFRs and CBRs demonstrated this pattern. For instance, rates of 137.13 and 33.16 births per 1,000 person-years, respectively were recorded for 2005, dropping to 127.06 and 30.57 births per 1,000 person-years by 2008, but increasing again in 2009.

#### Mortality

**Fig. 5:**
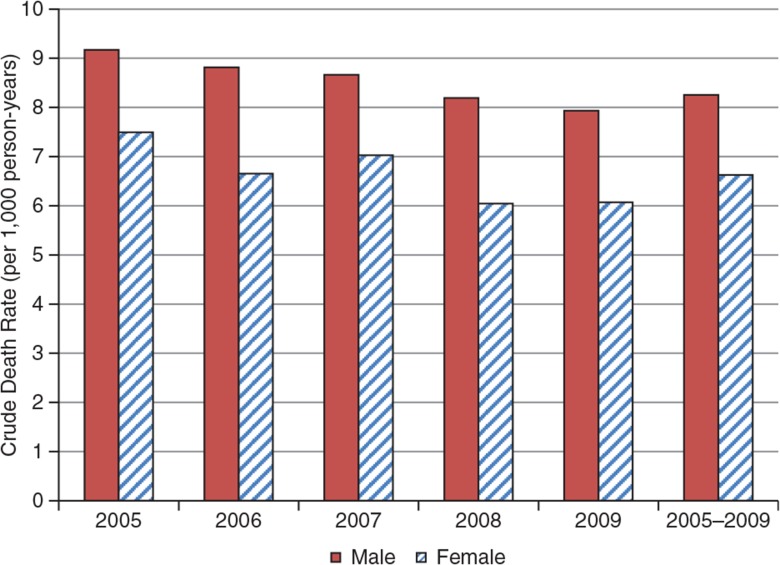
Crude death rates for KHDSS by sex, 2005–2009.

Figure 5 shows the CDRs of the KHDSS from 2005 to 2009. Overall, 4,722 deaths were recorded over the period 2005–2009 with the average number of deaths per year observed for the period being approximately 950 and the CDR approximately eight deaths per 1,000 person-years.

In general, the rate was higher for men than for women for all the years under consideration. For example, there was close to two more deaths among men than for women for every 1,000 person-years observed. However, there appears to be a slight decline for both sexes over the period.

**Fig. 6:**
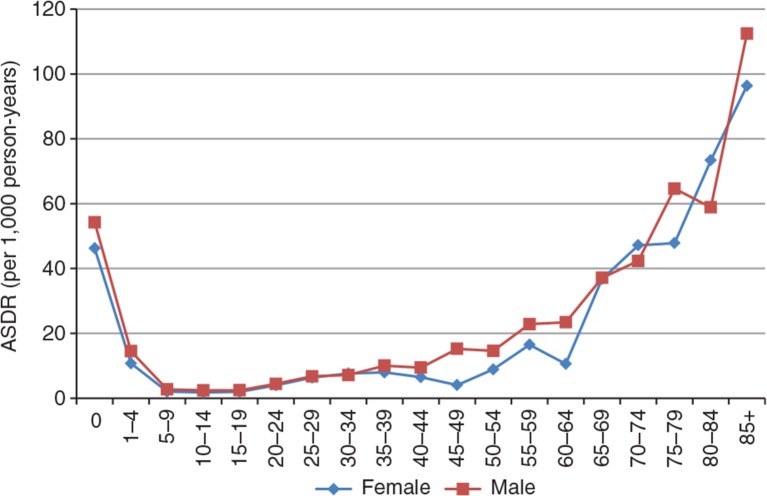
Age-specific death rates by sex, 2009.

As shown in Fig. 6, and also suggested by the crude death estimates, mortality levels were higher for men, although the age pattern of mortality is typical of most human populations. There were approximately 1.5 deaths per 1,000 person-years for 10–14 year-olds, to a mean of approximately14 per 1,000 person-years for 55–59 years, varying widely for both sexes. Old age mortality was relatively higher, as might be expected, with more than 90 deaths per 1,000 person-years for men aged 85 years or more.

**Fig. 7:**
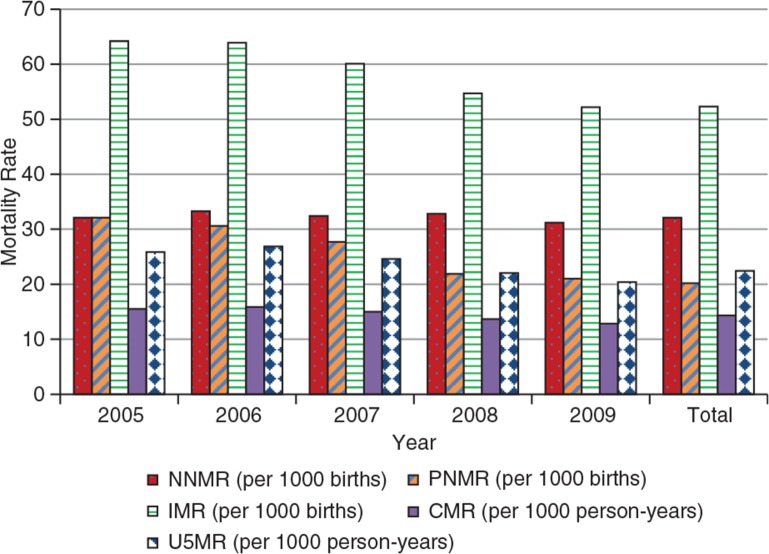
Neonatal, post-neonatal, infant, and child mortality rates for KHDSS, 2005–2009.

With respect to infant and child mortality levels, there was a reduction in infant mortality rates, mainly because of reductions in post-neonatal rates. Figure 7 shows the neonatal, post-neonatal, infant, and child mortality rates (children aged 1–4 years) for KHDSS over the period 2005–2009.

There were 32 neonatal deaths for every 1,000 live births. Furthermore, 20 post-neonatal deaths per 1,000 births were recorded annually, corresponding to an infant mortality rate of approximately 52 deaths per 1,000 live births. Infant mortality rates dropped by approximately 12 infant deaths per 1,000 births within the period. This decline consists mainly of a reduction in post-neonatal mortality from approximately 32 per 1,000 births in 2005 to 21 per 1,000 in 2009. In contrast, neonatal mortality did not decline, largely remaining at over 30 deaths per 1,000 births. Mortality among 1–4 year-olds and under-five generally declined per 1,000 person-years. The general reduction in overall mortality and particularly infant and child deaths (deaths to children aged 1–4 years) translated into an increase in life expectancy. This is illustrated in Table 3, which shows the probability of dying at age 0 (q_0_) and life expectancy at birth (e_0_) for KHDSS by sex for 2005 and 2009.

**Table 3:** Probability of dying at age 0 (q0) and life expectancy at birth (e0) for KHDSS by sex, 2005 and 2008

	2005		2008	
		
Sex	q_0_	e_0_	q_0_	e_0_
Male	0.052	57.5	0.051	59.7
Female	0.048	59.4	0.044	61.7

Overall, life expectancy increased for both sexes from 2005 to 2008. Average life expectancy at birth for both sexes was 58 years in 2005, rising to 60 years by 2009. On average, women live approximately 2 years longer than men.

#### Migration

Migration levels observed in the KHDSS are shown in Table 4.

**Table 4:** Migration rates^a^ per 1,000 person-years for KHDSS by year, 2005–2009

Year	In-migration rate	Out-migration rate	Gross migration rate	Net migration rate
2005	45.85	92.77	138.63	−46.92
2006	68.07	84.87	152.94	−16.80
2007	65.69	75.31	141.00	−9.63
2008	62.42	84.35	146.77	−21.94
2009	61.82	63.10	124.90	−1.29
Total	61.04	79.67	140.70	−18.64

a Gross migration rates are computed as the sum of all in- and out-migrations over the respective person-years; net migration rates are computed as the difference between in- and out-migrations over the sub-group population.

**Fig. 8:**
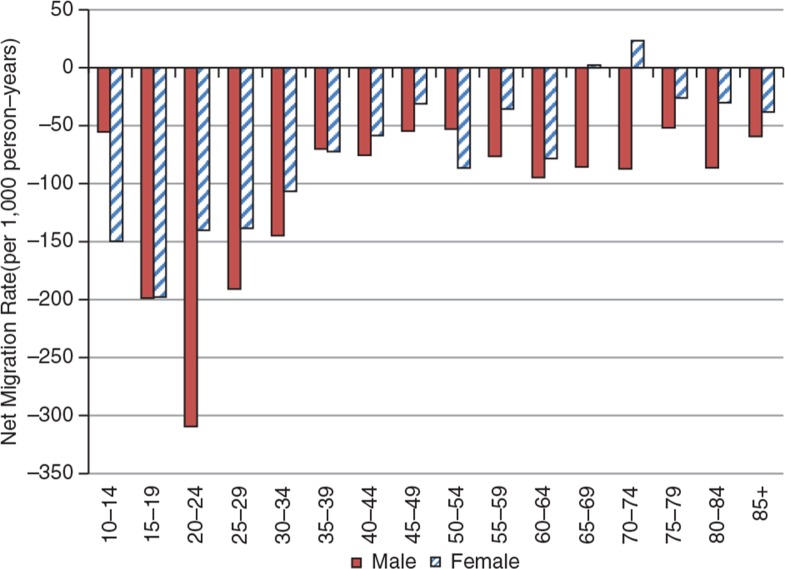
Age-specific net migration rates for KHDSS, 2005–2009.

Over the period, the surveillance area experienced some loss of population of approximately 20 excess out-migrations per 1,000 person-years, indicated by the total net migration rate. This deficit varied by year, from a peak of approximately 50 out-migrations in 2005 to approximately 1 out-migration per 1,000 person-years in 2009. As might be expected, the highest levels of out-migrations were recorded among the labour force population aged between 15 and 59 for all years, with the peaks at ages 20–24, as shown in Figs 7 and 8.

Over the period, there was a deficit in migrations for all age-groups, except for a small gain of approximately 20 migrations per 1,000 person-years observed for ages 65–74. However, this gain was insufficient to reverse the overall migratory deficit. Figure 8 shows the GMRs for the KHDSS for the period 2005–2009.

The general pattern of all migrations shows a rise from age range 10–14 to a peak at ages 20–24, and thereafter a decline for all age-groups with the lowest among the aged population. This is indicative of a relationship between age and number of migratory episodes.

#### Educational attainment

As at 2008, approximately half of the population (51.97%) had had no formal education. Furthermore, 23.76% had had primary education, 15.22% with middle/junior secondary, and 5.45% with secondary or higher education. Table 5 shows educational attainment of the KHDSS population by sex.

**Table 5:** Educational attainment for KHDSS by sex, 2008

Gender	No education (%)	Pre-school (%)	Primary (%)	Middle/JHS (%)	Secondary+ (%)
Male	48.6	3.7	24.3	16.1	7.3
Female	55.2	3.5	23.2	14.3	3.7

Table 5 suggests some gender variation in educational attainment. A higher proportion of females have no education, as compared with males. However, educational attainment for those who have ever been to school or are in school is similar at pre-school and primary level for both sexes. Gender disparities become more apparent as one climbs the academic ladder, with less females attending junior high school or senior high school, as shown in Table 6.

**Table 6:** School enrolment for KHDSS by age and sex

Age	Sex	In school (%)	Not in school (%)
6–11	Male	55.02	44.98
6–11	Female	54.80	45.20
12–18	Male	78.04	21.96
12–18	Female	75.08	24.92

#### Socio-economic and wealth access

Table 7 shows fertility and mortality rates by wealth quintile. In relation to demographic outcomes, the first two quintiles have lower fertility as well as lower mortality, compared to the last three quintiles.

**Table 7:** KHDSS fertility and mortality rates by wealth quintile

Quintile	General fertility rate	Total fertility rate	Crude death rate	Infant mortality rate (1q0)	Child mortality rate (4q1)	Under-Five mortality rate (5q0)	Crude death rate
First	82.45	2.62	4.74	20.59	6.52	27.11	4.74
Second	103.85	3.38	6.95	32.5	6.07	38.5	6.95
Third	126.32	4.06	7.54	45.52	12.86	58.38	7.54
Fourth	134.52	4.42	7.33	42.16	12.45	54.55	7.33
Fifth	138.94	4.64	8.20	54.33	12.27	66.41	8.20

## Discussion

Overall, the results show an increase in population of 14.5% from 2005 to 2009. A key factor for this increase was not necessarily an increase in growth rate but rather an increase in the number of communities under surveillance from 129 to 156, as a result of improvements in the motorable road network over the years (4). Furthermore, populations living in areas under surveillance but who had not been previously counted were now also enumerated. Births were the primary determinant of the population growth observed, since migrations did not have an overall positive effect on the surveillance population. If the new KHDSS communities were not considered, we would have observed an estimated annual growth rate of approximately 1%. At this rate, these communities are expected to double in 70 years from the 2005 estimate.

The population structure is typical of one found in many LDCs, with approximately 45% of the population under the age of 15 and this contrasts with approximately 17% in the developed countries (15). The elderly population is also undergoing a slight increase, as has been observed. Thus, there are approximately 90 dependents (aged less than 15 and 60 or more) for every 100 persons within the working-age group (15–59). The number of dependents for a working person has wide-ranging implications for poverty, including limited capital accumulation and even health status. In reality, this ratio may be even larger for the KHDSS, considering that a proportion of the 15–59 year-olds are out of work and therefore also dependent on those employed.

The population pyramid also suggests a deficit of infants, most likely due to under-reporting. Other contributing factors may be the periodicity of follow-up and misclassification of live births as in-migration. Households are visited every 6 months, and if pregnancies are not disclosed at first contact due to cultural and/or other reasons, including pregnancies missed by a fieldworker for registration, a pregnancy outcome of birth, or a neonatal death shortly afterward may also be missed.

**Fig. 9:**
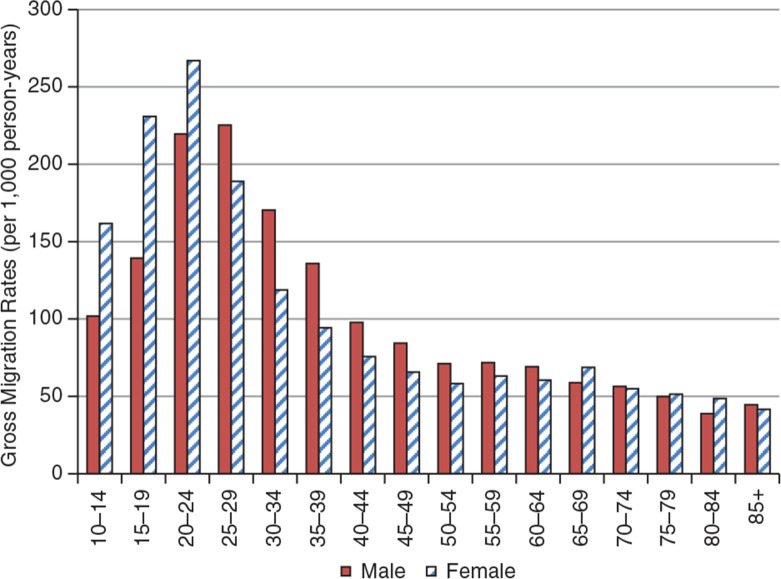
Age-specific gross migration rates for KHDSS by sex, 2005–2009.

There is an excess of males to females within the teen years, which is reversed from age 20 up to 49 years and again reversed to a deficit of females for ages 50–69; in the age group 70 and above, there are more females. The observed patterns are due to migration and mortality. The deficit of females in their teens and males aged 20–49 is primarily due to migration outside the KHDSS area, whilst the excess of females over males in the older age group could be attributed to mortality, since at that age band migration is very low (see Fig. 9). There is some anecdotal evidence to suggest that Brong-Ahafo Region is a major source of irregular migrations outside the country. However, further studies need to be undertaken to establish the levels and patterns of irregular migrations in the KHDSS area.

Single-year population pyramids (not shown) also demonstrate digit preference of zero and five at ages above 10. Digit preference refers to the tendency of respondents to report ages ending in digit ‘0’ or digit ‘5’, particularly where their true ages are not known. However, the ages of children born after the start of the KHDSS are more accurate since dates of birth are well documented with the use of the identification system which records the date of birth and facilitate births registration with the BDR department by the HDSS staff.

As might be expected, a largely rural population, with low levels of education and limited household wealth, underpinned the observed population growth. Estimates from the 2008 GDHS showed that one in four respondents from the Brong-Ahafo Region had no education. This contrasts significantly with the KHDSS area where close to half of all residents in reproductive age (15–49 years) has no education. Coupled with a large dependency ratio and widespread poverty, the ability to build up property or funds for investment is limited.

In some LDCs, particularly in East Asia, young populations have fewer children than previous generations as they enter adulthood, increasing the number of working-age adults and reducing youth dependency (15). This demographic dividend has accelerated economic growth, as significant fertility decline facilitated high levels of education, especially among females, translating into increased productivity. Although female education is improving in the KHDSS with approximately 9% of 15–30 year-olds having secondary or higher education, demographic dividends may not be reaped imminently, as dependency levels remain high and the availability of professional skill remains limited within the population. Close to half of the 6–11 year-olds (primary school age) and a quarter of 12–18 year-olds (secondary school age) who should be in school are not. This is a key challenge, especially if MDG 2, which calls for boys and girls everywhere to complete a full course of primary schooling, is to be met. To harvest demographic dividends, there is a need to provide high quality and accessible education and health services to large numbers of young people. Ultimately, the prohibitive costs involved will make this unattainable for the surveillance area and for the nation at large.

TFRs in the KHDSS for the period do not differ much from national and regional estimates of 4.0 and 4.1, respectively (16). Births were the main driving force of growth. The slight reduction in fertility occurred concurrently with one observed nationally between 2003 and 2008. The national TFR dropped from 4.4 to 4.0, whilst that of the Brong-Ahafo Region also dropped from 4.8 to 4.1. Indeed, there is a need for more work to be done before fertility reaches replacement level, as there was a resurgence in fertility in 2009. Approximately one in 10 births in the KHDSS was to a teen mother. The associated dangers for both the mother and the child are obvious. These include pregnancy-related complications and death, possible abortions, disrupted education, greater lifetime fertility, increased risk of HIV, and other sexually transmitted infections (STIs) as well as a life of poverty and the added pressures they exert on the health delivery system (17, 5). The drastic drop in fertility after age 39 years was very similar to the national pattern.

On average, 32 neonatal deaths were registered for every 1,000 live births and this occurred annually from 2005 to 2009. Furthermore, approximately 52 infant deaths per 1,000 live births were registered in the KHDSS, which were largely in agreement with GDHS 2008 estimates. The KHDSS, however, recorded significantly lower child mortality compared to GDHS 2008 estimates (14 child deaths per 1,000 person-years and 31 deaths per 1,000 children, respectively. MDG 4 calls for reducing by two-thirds the mortality rate of children under the age of 5 years by 2015. Together with Goal 5, (which calls for reducing maternal mortality ratio by three-quarters by 2015) these are perhaps the two most popular MDGs in Ghana. Within the KHDSS area as well as nationally, they have attracted a lot of attention, with interventions such as improvements in antenatal services and treatment of pregnancy-related malaria targeted towards them. Within the KHDSS area, under-five mortality remained at approximately 22 deaths per 1,000 person-years. This was made up largely of infant deaths, particularly at neonatal stage. With the decline of infant mortality from 2005 to 2009, under-five mortality declined by approximately 4% from 2006 to 2009.

CDRs are also declining within the KHDSS, a phenomenon observed in many LDCs (18). There is improved access to health care, as more people enrol for health insurance. Also, more people are better informed about their health and the need to take prompt action concerning illness. As this translates into rising life expectancy, the implication for population structure is an increasing proportion of the elderly. This is expected to lead to an increase in demand for treatment for chronic diseases, mental health problems and services such as social security for the elderly. As fertility levels are not declining as fast as mortality, natural increase is expected to pertain for the foreseeable future. This article has shown that fertility and mortality levels in the KHDSS could be influenced by socio-economic and wealth factors, as is to be expected. A reduction in fertility and mortality levels is, therefore, linked with the overall socio-economic development of the population.

On the whole, the Kintampo districts appear to have high levels of migration. Indeed, it is a net exporter of labour, with an average annual deficit of 24 out-migrations per 1,000 person-years. The population structure of the KHDSS also indicates a deficit of female population within the teen years of 901.94 female person-years to every 1,000 male person-years among 10–19 year-olds. It is fair to assume that a certain proportion of these movements may be related to out-migrations for some form of labour outside the KHDSS. There should be a similar explanation for the observed shortage of men from age 20 to 39 on the population pyramids. This is symptomatic of a youthful population that continues to grow rapidly, but with few opportunities for education and employment to match the level of growth. These migration patterns also influence fertility and mortality indicators in that they are selective in the age-groups involved. These may also account for differences between outputs from areas with demographic surveillance and those from national censuses and surveys. These migration patterns also influence fertility and mortality indicators in that they are selective in the age-groups involved. These may also account for differences between outputs from demographic surveillance and those from censuses and surveys. National statistics as they relate to the surveillance area can be adjusted, using rates from the demographic surveillance systems. The middle belt statistics can be corrected using the KHDSS rates generated. This same approach can be used for the statistics in the southern belt where Dodowa HDSS is located and northern belt where Navrongo HDSS is also located. Triangulating these data sources takes advantage of their unique strengths, that is, the representativeness of national data and the longitudinality of surveillance data.

The benefits of the KHDSS extend beyond the estimation of various demographic events. Establishing the KHDSS has brought up several new opportunities as we are able to carry out large population level surveys and in specific risk groups and determine emerging health and social problems. In the last few years, KHDSS updates have included modules on a number of health issues. For instance, the KHDSS has collected data on biomass cooking practises and how they influence health, knowledge, and behaviour related to tuberculosis and HIV, use of insecticide treated nets, artemisinin combination therapies, and intermittent preventive treatment of malaria in pregnancy among pregnant women. Data on causes of death are also an important component of KHDSS data collection. These data are often non-existent in LDCs, thus providing valuable information for policy. Considering that research infrastructure is already in place in the KHDSS, it is important that health authorities utilise these capabilities by making use of the KHDSS as a sentinel site. Thus, findings from previous interventions can be made available more widely, so that it is not just the demographic surveillance area (DSA) that benefits.

In spite of its advantages, the KHDSS does not have core funding. It depends on projects that use the KHDSS framework for their own data collection. The effects are that funding dries up when projects end. Also, coding of cause of death data is delayed pending funds, and periodicity of routine updates is longer. Indeed, strengthening HDSSs could mainstream this vital resource to evidence-driven policy making at the local level (19). By themselves, the HDSSs in Ghana (i.e. Navrongo, Kintampo, and Dodowa) may lack representativeness of the country. However, with data pooled together, they provide important insights for the whole country, as each is more representative of the ecological and socio-economic area in which it is found. Being a longitudinal surveillance system, the trends over time, as documented, will serve as a good guide in planning for interventions in the country. This becomes prominent for indicators that are worsening over time.

## Conclusion

The dearth of health and demographic data in sub-Saharan Africa from vital registration systems and its impact on effective planning for health and socio-economic development is widely documented. HDSSs have, therefore, played a crucial role in providing more current information on health and demographic patterns and trends in developing countries. The KHDSS population is youthful, with a growth rate of 1% per annum. With an inbuilt population momentum of children entering reproductive age, population is expected to grow in the foreseeable future. Recent fertility decline was not sustained in 2009, though mortality declined slowly, leading to a rise in life expectancy. However, it is unlikely that there would be demographic dividend in the near future. Also, the KHDSS is a net exporter of people in the working-age group through out-migration. The net loss of teen females and the prevailing level of teen fertility may require further studies to unravel the related factors and inform policy on interventions.

## References

[1] Byass P, Berhane Y, Emmelin A, Kebede D, Andersson T, Hogberg U (2002). The role of demographic surveillance systems (DSS) in assessing the health of communities: an example from rural Ethiopia. Public Health.

[2] Byass P, Kahn K, Fottrell E, Collinson MA, Tollman SM (2010). Moving from data on deaths to public health policy in Agincourt, South Africa: approaches to analysing and understanding verbal autopsy findings. PLoS Med.

[3] INDEPTH Network (2002). Population and health in developing countries, volume 1: population, health and survival.

[4] Nettey EA, Zandoh C, Sulemana A, Adda R, Owusu-Agyei S (2010). Clustering of childhood mortality in the Kintampo Health and Demographic Surveillance System. Global Health Action.

[5] United Nations (1994). Programme of action adopted at the International Conference on Population and Development.

[6] Finkle JL, McIntosh CA (2002). United Nations Population Conferences: shaping the policy agenda for the twenty-first century. Stud Fam Plann.

[7] Salawu B (2009). Strengthening vital registration systems as a source of demographic data for effective socio-economic development planning in Nigeria. Pak J Soc Sci.

[8] Mathers CD, Ma Fat D, Inoue M, Rao C, Lopez AD (2005). Counting the dead and what they died from: an assessment of the global status of cause of death data. Bull World Health Organ.

[9] ICF Macro (2010). Trends in demographic, family planning, and health indicators in Ghana, 1960–2008: trend analysis of demographic and health surveys data. DHS Trend Report No. 6.

[10] National Population council (1994). Government of Ghana National Population Policy.

[11] Sankoh O, Byass P (2012). The INDEPTH Network: filling vital gaps in global epidemiology. Int J Epidemiol.

[12] Serwaa-Bonsu A, Herbst AJ, Reniers G, Ijaa W, Clark B, Kabudula C (2010). First experiences in the implementation of biometric technology to link data from Health and Demographic Surveillance Systems with health facility data. Glob Health Action.

[13] Bangha M, Diagne A, Bawah A, Sankoh O (2010). Monitoring the Millennium development goals: the potential role of the INDEPTH Network. Glob Health Action.

[14] Sankoh O, Binka F, Becher H, Kouyate B (2005). INDEPTH Network: generating empirical population and health data in resource-constrained countries in the developing world. Health research in developing countries: a collaboration between Burkina Faso and Germany.

[15] Population Reference Bureau (2010). Population Bulletin.

[16] Ghana Statistical Service (GSS), Ghana Health Service (GHS), and ICF Macro (2009). Ghana.

[17] Bernstein S (2005). Population, reproductive health and the millennium development goals: messages from UN Millennium Project reports.

[18] McMichael AJ, McKee M, Shkolnikov V, Valkonen T (2004). Mortality trends and setbacks: global convergence or divergence. Lancet.

[19] Baiden F, Hodgson A, Binka FN (2006). Demographic Surveillance Sites and emerging challenges in international health. Bull World Health Organ.

